# ASSOCIATION BETWEEN PARTNER IDENTIFICATION AND EDUCATION WITH HOSPITAL LENGTH OF STAY FOR PATIENTS WITH DEMENTIA

**DOI:** 10.1093/geroni/igae098.0660

**Published:** 2024-12-31

**Authors:** Nicole Werner, Austin Medlin, Beth Fields

**Affiliations:** Indiana University, Bloomington, Bloomington, Indiana, United States; Indiana University Bloomington, Bloomington, Indiana, United States; University of Wisconsin Madison, Madison, Wisconsin, United States

## Abstract

Despite overwhelming reliance on care partners to care for people living with dementia (PLWD) after a hospitalization, care partners rarely receive adequate education to meet post-hospitalization caregiving demands, which is associated with health consequences including adverse clinical outcomes for PLWD, and increased levels of stress, depression, and morbidity for care partners. The present study aimed to explore associations between care partner identification as documented in the electronic health record (EHR), documented care partner education, and PLWD length of stay (LOS). We extracted EHR data from a large academic medical center in the Midwest between 1/1/2019 and 8/1/2022 (N = 8038). To assess association between care partner identification and PLWD LOS, encounters were coded dichotomously, either ‘lacking a care partner’ or ‘care partner present’, depending on EHR documentation. Similarly, encounters received designations of increased education if care partners received more education on post-discharge care than the education sample median. Independent t-tests analyzed mean differences on LOS for care partner identification. The same approach was used on a subsample of encounters with care partner identification (n=1644) to analyze associations between care partner education and LOS. We found no significant difference in LOS based on care partner identification status (p=.072). Among PLWD with identified care partners, significant differences in LOS were found between education groups (p=.017), where longer hospitalizations were associated with more care partner education (less education M=5.9 days, more education M=6.9). These results suggest that targeted education could better support care partners when a shorter LOS is expected for PLWD.

